# Inhibition of HSPA8 alleviates experimental autoimmune encephalomyelitis via dual modulation of NLRP3 inflammasome activation: suppressing both NF-κB–mediated priming and ASC-dependent assembly

**DOI:** 10.1038/s41420-026-03215-7

**Published:** 2026-06-24

**Authors:** Qianqian Bai, Yan Guo, Shunji Pan, Fangfei Liu, Wenchao Tang, Xiaozhi Bai, Shumin Zhang, Xiaoyu Ma, Qizhi Fu, Dongmei Wang, Hua Fan

**Affiliations:** 1 The First Affiliated Hospital, and College of Clinical Medicine of Henan University of Science and Technology, Luoyang, China; 2 Department of Intensive Medicine, The First Affiliated Hospital, and College of Clinical Medicine of Henan University of Science and Technology, Luoyang, China; 3 School of Basic Medical Sciences, Henan University of Science and Technology, Luoyang, China; 4 Luoyang Key Laboratory of Neuroimmunology and Innovative Drug Screening, Luoyang, China; 5 Office of Research & Innovation, The First Affiliated Hospital, and College of Clinical Medicine of Henan University of Science and Technology, Luoyang, China

**Keywords:** Multiple sclerosis, Neurodegenerative diseases, Inflammasome

## Abstract

The pathological processes of multiple sclerosis (MS) and its animal model, experimental autoimmune encephalomyelitis (EAE), are closely associated with excessive activation of inflammasomes. HSPA8 is a constitutively expressed molecular chaperone involved in cellular signaling and immune-inflammatory regulation, but its role in EAE-associated neuroinflammation remains unclear. In this study, we found that HSPA8 was significantly upregulated in the spinal cord tissues of EAE mice and positively correlated with disease severity. Immunofluorescence co-staining showed that HSPA8 upregulation was more prominently associated with Iba1-positive microglia/macrophage-enriched regions than with GFAP- or NeuN-positive regions. Intrathecal knockdown of HSPA8 attenuated EAE progression, reduced inflammatory responses, and alleviated demyelination and axonal injury. In vitro, HSPA8 knockdown reduced NLRP3 inflammasome-mediated IL-1β/IL-18 release, caspase-1 activation, GSDMD-N formation, and pyroptosis in THP-1 cells, bone marrow-derived macrophages, and BV2 microglia, and these effects were partially restored by siRNA-resistant HSPA8 rescue. Mechanistically, HSPA8 knockdown was associated with impaired NF-κB-dependent priming, as reflected by reduced p65 phosphorylation, nuclear translocation, NF-κB transcriptional activity, and pro-IL-1β expression. HSPA8 is also associated with ASC, and HSPA8 knockdown impaired NLRP3–ASC interaction, ASC oligomerization, and ASC speck formation during inflammasome assembly. These findings suggest that HSPA8 is functionally associated with NLRP3 inflammasome activation through both NF-κB‑dependent priming and ASC‑associated assembly, thereby contributing to neuroinflammation in EAE. HSPA8 may represent a potential therapeutic target for MS-related and other inflammasome-driven neuroinflammatory disorders.

## Background

Multiple sclerosis (MS) is an autoimmune inflammatory disorder that impacts the central nervous system (CNS). Its characteristic pathological changes include demyelination, axonal degeneration, and neuronal damage, accompanied by extensive immune cell infiltration. MS is currently one of the most significant causes of nontraumatic disability among young adults (aged 20–40 years) [1, 2]. Although immunomodulatory agents (e.g., interferon-β, fingolimod, and monoclonal antibodies) have significantly improved the recurrence rate and short-term prognosis of MS in recent years, these treatments still cannot effectively prevent the condition from evolving into progressive MS [2–5]. The fundamental reason lies in the highly complex pathogenesis of MS, which is still not completely understood. Animal models of experimental autoimmune encephalomyelitis (EAE) are universally regarded as ideal for studying the pathogenesis and treatment strategies of MS, owing to their high similarity to the biochemical, immunological, and pathological features and the clinical manifestations of MS [6]. Therefore, exploring the molecular mechanisms and intervention methods for MS using EAE models holds immense research significance in the prophylaxis and treatment of MS.

Recent research has revealed the crucial role of the abnormal activation of the innate immune system in the pathology of MS/EAE. Specifically, the inflammatory responses mediated by microglia in the CNS and macrophages infiltrating the periphery constitute the core pathological link driving myelin loss and axonal damage. Inflammasomes, crucial components of innate immunity, sense danger or stimulus signals and mediate a cascade of inflammatory responses, serving as important “sensors” and “effectors” of the innate immune system [7]. Among the various inflammasomes that have been discovered, NOD-like receptor pyrin domain–containing protein 3 (NLRP3) is considered to be closely related to the onset of MS. Several clinical studies have reported abnormal increases in various components of the NLRP3 inflammasome proteins, such as NLRP3, apoptosis-associated speck-like protein containing a CARD (ASC), and caspase-1 in the cerebrospinal fluid and serum of patients with MS [8–12]. Emerging evidence shows that NLRP3 inflammasome activation triggers caspase-1, leading to the release of potent pro-inflammatory factors, notably interleukin (IL)-1β and IL-18. These cytokines promote the activation and infiltration of T cells (especially Th1/Th17) and B cells, thereby amplifying the neuroinflammatory cascade. This process ultimately results in demyelination, axonal damage, and neuronal loss [8, 13]. Additionally, activated NLRP3 contributes to MS pathogenesis through multiple parallel pathways: it directly induces pyroptosis via Gasdermin D (GSDMD) cleavage, inhibits oligodendrocyte differentiation and remyelination, exacerbates blood-brain barrier disruption, and promotes the formation of reactive oxygen species-dependent neutrophil extracellular traps [12, 14]. Furthermore, NLRP3 inhibitors such as MCC950 [15], OLT1177 [16], and dimethyl fumarate [17, 18], as well as the gene knockout of key components such as NLRP3 and ASC [19], can significantly improve the symptoms of MS/EAE by limiting aberrant NLRP3 inflammasome activation. Collectively, these findings support the NLRP3 inflammasome as a key driver of MS/EAE pathology and a promising therapeutic target.

Heat shock protein A8 (HSPA8, also known as HSC70) is a constitutively expressed and highly conserved molecular chaperone. Its roles in fundamental processes such as protein folding, transport, degradation, and autophagy are well-documented [20, 21]. Recent evidence has revealed that the role of HSPA8 is not limited to being a “molecular chaperone.” It plays a complex regulatory role in various pathophysiological processes via specific protein-protein interactions. For instance, HSPA8 interacts with macrophage-expressed NLR family CARD domain-containing 5 (NLRC5) to alleviate pathological cardiac remodeling [22]. More importantly, studies in non-CNS disease models have demonstrated that targeting HSPA8 can modulate tissue injury by inhibiting aberrant NLRP3 inflammasome activation in conditions such as spinal cord ischemia-reperfusion injury and acute pancreatitis-associated lung injury [23–25]. These observations collectively suggest a potential functional connection between HSPA8 and NLRP3-driven inflammation. Furthermore, autoantibodies against HSP70 have been identified in the cerebrospinal fluid of individuals diagnosed with MS [26]. These discoveries suggest that HSP family members may be involved in autoimmune responses within the CNS of patients with MS. Nevertheless, the specific biological function and molecular mechanism of HSPA8 in autoimmune demyelinating diseases such as MS/EAE remain unclear.

This study aimed to determine whether and how HSPA8 modulates neuroinflammation in EAE through regulation of the NLRP3 inflammasome. Here, we report that HSPA8 expression is significantly upregulated in the spinal cords of EAE mice, and that HSPA8 knockdown markedly alleviates the clinical symptoms and histopathological changes of EAE. Mechanistically, our findings suggest that HSPA8 is functionally associated with both NF-κB-dependent priming and ASC-dependent assembly of the NLRP3 inflammasome. Our work supports a pathogenic role for HSPA8 in EAE and proposes a dual regulatory model of inflammasome activation, providing a foundation for exploring HSPA8 as a potential therapeutic node in MS and other inflammasome‑driven neuroimmune disorders.

## Results

### HSPA8 expression is upregulated in mice with EAE, and HSPA8 knockdown significantly attenuates disease progression in model mice

**Fig. 1:**
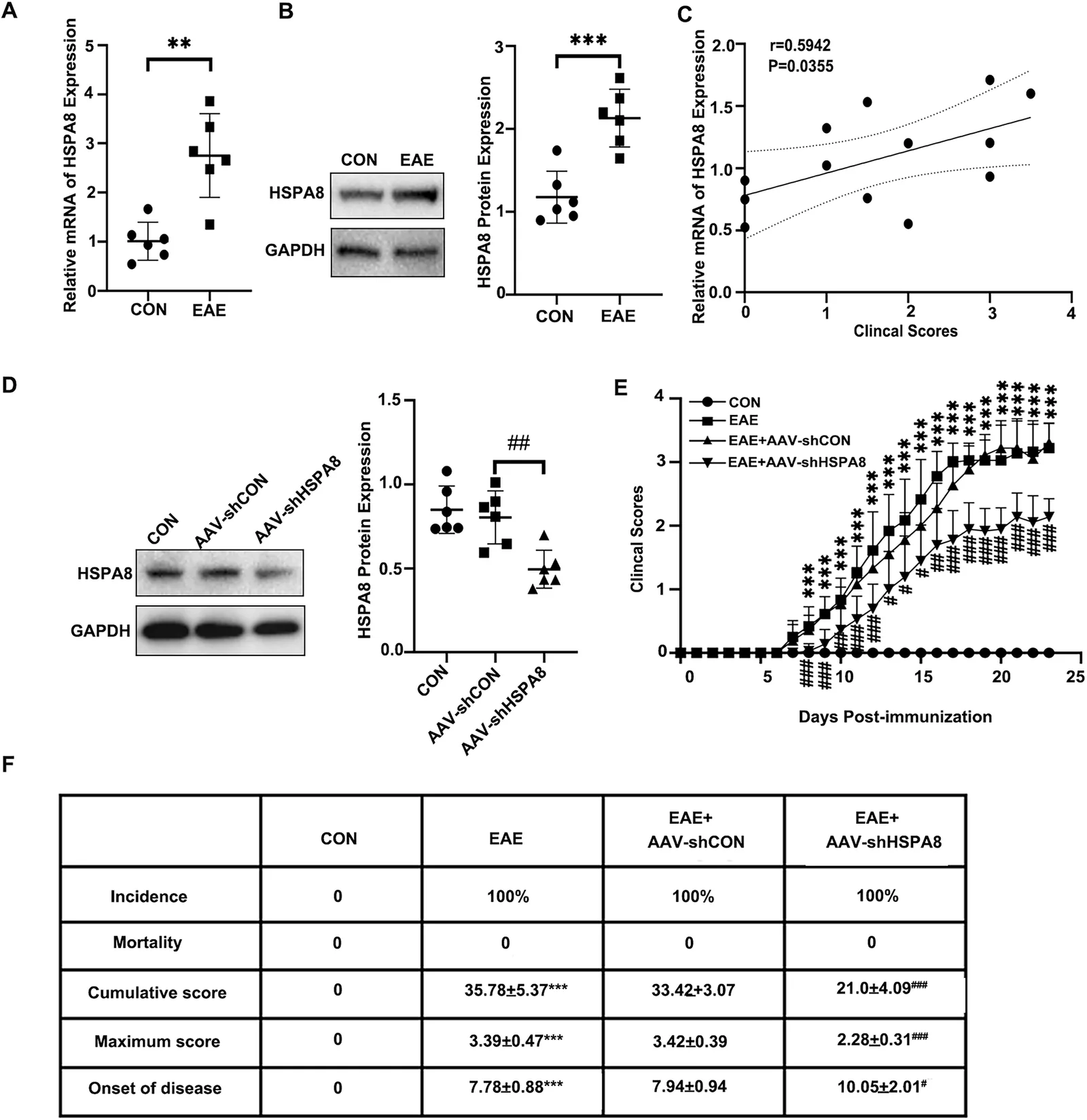
HSPA8 knockdown ameliorated the progression of EAE. HSPA8 expression was upregulated in the spinal cords of EAE mice at the mRNA (**A**, qPCR) and protein (**B**, Western blot) levels (*n* = 6). **C** Correlation between HSPA8 expression levels in the spinal cord of EAE mice and disease severity (clinical score) (*n* = 13). **D** HSPA8 was knocked down in mice by intrathecal injection of an adeno-associated virus (AAV-shHSPA8), as confirmed by Western blot (*n* = 6). **E** Clinical scores from day 0 to day 23 were recorded and analyzed (*n* = 18). **F** Quantitative analysis of cumulative scores, maximum scores, and disease onset day (*n* = 18). Data are presented as mean ± SD. ns, statistically insignificant. **P < 0.01, ^***^P < 0.001 vs. CON. ^*#*^*P* < 0.05, ^*##*^*P* < 0.01, ^*###*^*P* < 0.001, vs. EAE + AAV-CON. For A and B, an unpaired two-sided Student’s *t*-test was used. After testing for normality, variables C were analyzed using the Pearson correlation coefficient (r) (for normally distributed data). For F, one-way ANOVA followed by Tukey’s multiple comparison test was used. E was analyzed through two-way repeated measures ANOVA with post hoc Bonferroni multiple comparison test.

To explore HSPA8’s role in the pathogenesis of MS, we utilized the EAE mouse model. At the disease peak, spinal cords were harvested from euthanized mice. HSPA8 expression was analyzed by qPCR and Western blot. Compared with healthy controls, EAE mice exhibited a significant upregulation of HSPA8 at both the mRNA (Fig. 1A) and protein (Fig. 1B) levels in spinal cord tissues. Furthermore, HSPA8 expression levels in the spinal cord of EAE mice and disease severity (clinical score) showed a marked positive correlation (Pearson r = 0.5942, *P* = 0.0355) (Fig. 1C).

To define HSPA8’s functional role in EAE, we knocked down its expression via intrathecal injection of an AAV carrying an HSPA8-specific shRNA (efficiency verified in Fig. 1D). Mice exhibited clinical symptoms of EAE at days 7–8 after immunization, peaking at days 17–21. They presented with bilateral hind limb paralysis, and the cumulative and maximum clinical scores were 35.78 ± 5.37 and 3.39 ± 0.47, respectively (Fig. 1E, F). In contrast, HSPA8-knockdown mice exhibited significantly reduced symptoms, with cumulative and maximum clinical scores reducing to 21.00 ± 4.09 and 2.28 ± 0.31, respectively. The onset time was also delayed from 7.78 ± 0.88 days to 10.05 ± 2.01 days (Fig. 1E, F). Thus, HSPA8 was significantly upregulated in EAE mice, and HSPA8 knockdown could alleviate disease symptoms, supporting the involvement of HSPA8 in EAE progression.

### HSPA8 knockdown attenuates myelin loss in EAE mice

**Fig. 2:**
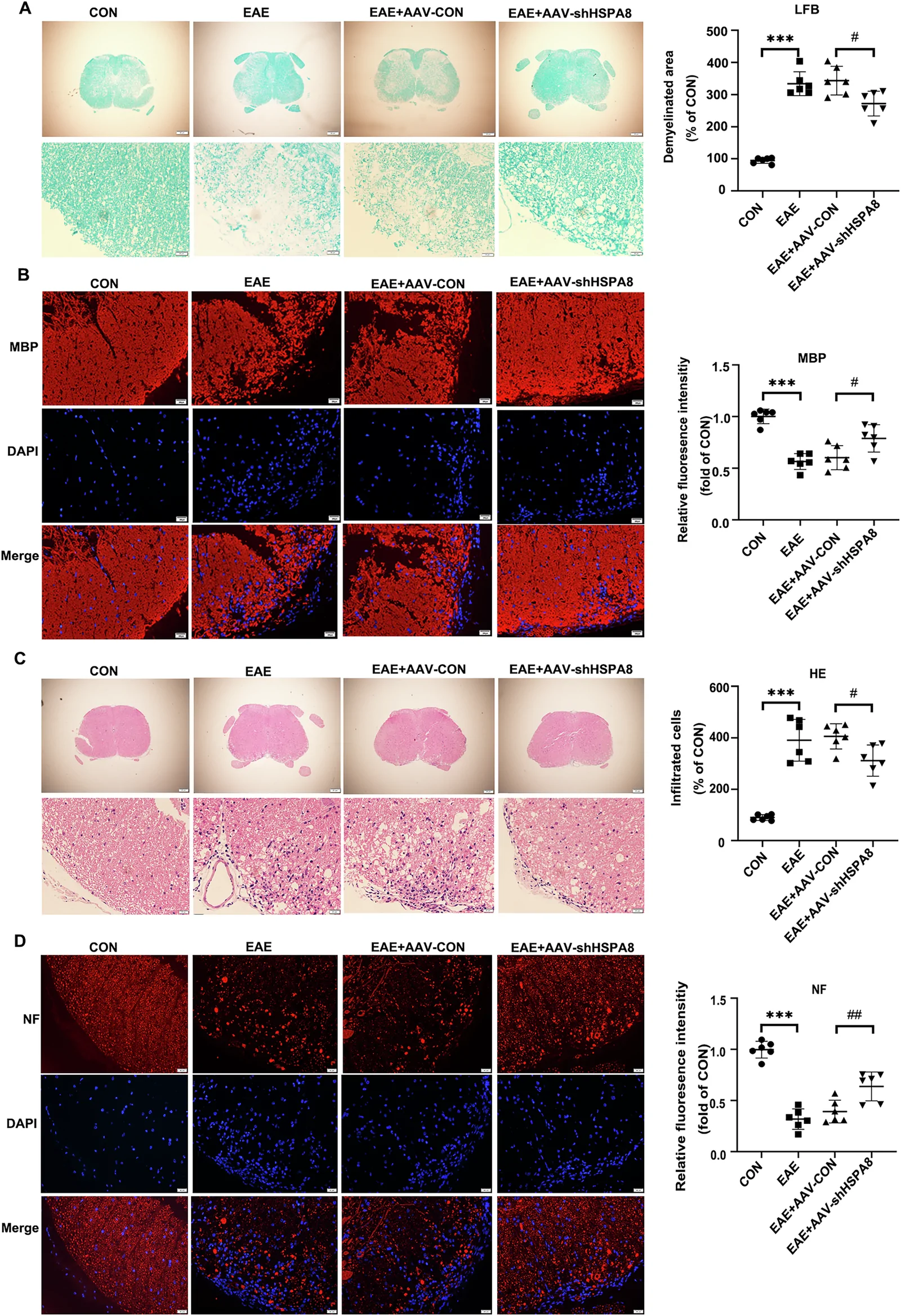
HSPA8 knockdown attenuates myelin loss, inflammatory cell infiltration, and axonal damage in EAE mice. Mice were administered AAV-shHSPA8 or control virus via intrathecal injection, followed by EAE induction. Spinal cord sections were then stained with LFB (**A**) or immunostained for MBP (**B**) to assess myelin integrity (*n* = 6). Scale bars: 50 μm (**A**); 200 μm (**B**). Spinal cord sections were stained with H&E (**C**) for inflammatory cells or with an anti-NF antibody (**D**) for axons (*n* = 6). Scale bars: 20 μm. Data are mean ± SD. ^***^P < 0.001 vs. CON. ^*#*^*P* < 0.05, ^*##*^*P* < 0.01, vs. EAE + AAV-CON. The **A**–**D** data were analyzed using one-way analysis of variance, combined with Tukey’s multiple comparison test.

After confirming that HSPA8 knockdown alleviated the clinical manifestations of mice with EAE, its effects on myelin degeneration and other neurohistopathological changes were further evaluated. As shown in Fig. 2A, LFB staining revealed intact and orderly morphology of the spinal cord in the normal control group, indicating no obvious myelin degeneration. Pathological sections of the spinal cords of EAE mice revealed point-like or even sheet-like white vacuolated areas of myelin loss, with these areas overlapping highly with the inflammatory cell infiltration areas of the section. AAV-CON administration before EAE modeling did not effectively reverse this outcome, whereas injecting AAV-shHSPA8 to exogenously downregulate HSPA8 expression significantly alleviated EAE-induced myelin degeneration.

To corroborate the demyelination observed by LFB staining, we assessed myelin integrity by immunofluorescence for MBP. Consistent with the LFB results, MBP fluorescence intensity in spinal cord sections was markedly decreased in EAE mice relative to control mice (Fig. 2B), displaying a discontinuous and fragmented pattern. This loss of MBP signal was not prevented by control AAV but was markedly preserved in the HSPA8-knockdown group (Fig. 2B).

### HSPA8 knockdown reduces inflammatory cell infiltration and axonal damage in EAE mice

To assess neuroinflammation and axonal integrity, spinal cord sections were analyzed by H&E staining and immunofluorescence. As shown in Fig. 2C, the spinal cord tissue sections of EAE mice exhibited extensive diffuse inflammatory cell infiltration, with some areas showing typical perivascular cuffing. It is noteworthy that before the establishment of the mouse model of EAE, the above situations could be significantly improved by injecting AAV-shHSPA8 to downregulate HSPA8 expression.

Furthermore, as shown in Fig. 2D, immunofluorescence staining of NF in the spinal cord tissues from the normal control group was uniform, and the axons showed normal arrangement without any obvious damage. After EAE modeling, NF signals were markedly reduced and became discontinuous, accompanied by axonal swelling, fragmentation, and the formation of axonal spheroids. HSPA8 knockdown notably preserved NF fluorescence intensity and alleviated EAE-induced axonal damage.

### HSPA8 knockdown attenuates inflammatory responses and NLRP3 inflammasome activation in EAE mice

**Fig. 3:**
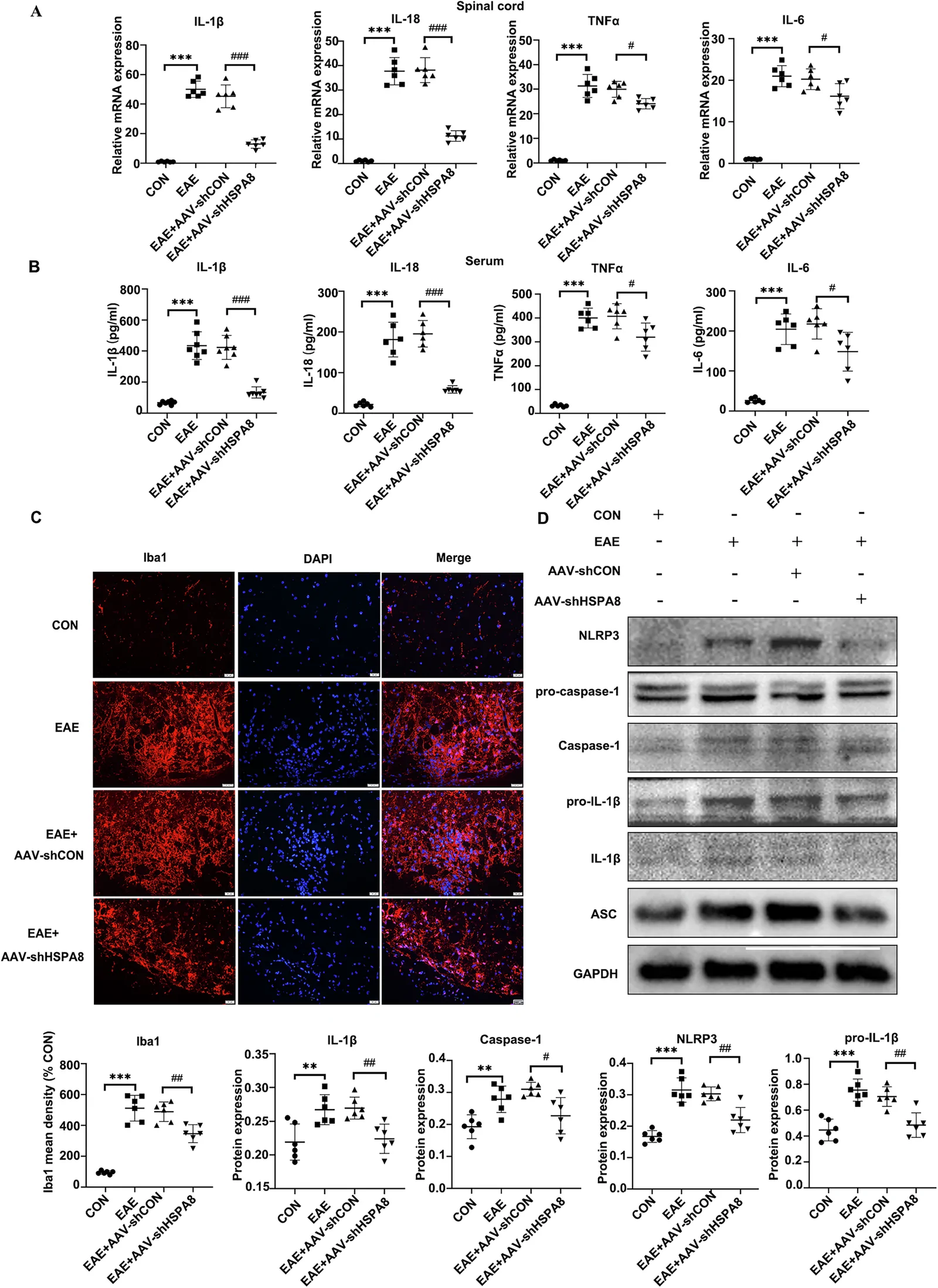
HSPA8 knockdown attenuates inflammatory responses and NLRP3 inflammasome activation in EAE mice. **A** qPCR analysis of cytokine mRNA in spinal cords (*n* = 6). **B** ELISA of cytokines in serum (*n* = 6). **C** Iba1 immunostaining of spinal cord microglia (*n* = 6). Scale bar: 20 μm. **D** Western blot analysis of NLRP3 pathway proteins in spinal cords (*n* = 6). Data are mean ± SD. ^**^P < 0.01, ^***^P < 0.001, vs. CON. ^*#*^*P* < 0.05, ^*##*^*P* < 0.01, ^*###*^*P* < 0.001, vs. EAE + AAV-CON. The data were analyzed using one-way analysis of variance, followed by Tukey’s multiple comparison test.

To elucidate how HSPA8 alleviates EAE, we first examined key pro-inflammatory cytokines. qPCR analysis revealed that mRNA levels of IL-1β, IL-18, TNF-α, and IL-6 were markedly elevated in the spinal cords of EAE mice (Fig. 3A). This upregulation was effectively reversed by HSPA8 knockdown. Consistent with this, ELISA detected a parallel increase of these cytokines in serum, which was also diminished upon HSPA8 knockdown (Fig. 3B).

Considering the core role of microglia/macrophages in neuroinflammation, we evaluated their activation via Iba1 immunofluorescence. EAE spinal cords exhibited a marked increase in Iba1⁺ cells, which displayed an activated, ameboid morphology (Fig. 3C). HSPA8 knockdown significantly reduced both microglial numbers and their activated phenotype (Fig. 3C).

To further assess the cellular distribution of HSPA8 in EAE spinal cords, we performed additional immunofluorescence co-staining of HSPA8 with markers for microglia/macrophages (Iba1), astrocytes (GFAP), and neurons (NeuN). HSPA8 expression was markedly increased in EAE spinal cords and showed more evident spatial overlap with Iba1-positive microglia/macrophage-enriched regions than with GFAP- or NeuN-positive regions (Supplementary Fig. 1). Considering that microglia/macrophage-lineage cells are major CNS innate immune effectors and that IL-1β and IL-18 were more prominently reduced after HSPA8 knockdown, we next examined whether NLRP3 inflammasome signaling was affected in EAE. Western blot analysis showed that the levels of caspase-1, IL-1β, NLRP3, and pro-IL-1β were significantly increased in EAE spinal cords, whereas their expression was markedly reduced following HSPA8 knockdown (Fig. 3D).

### HSPA8 knockdown attenuates NLRP3 inflammasome activation in vitro

**Fig. 4:**
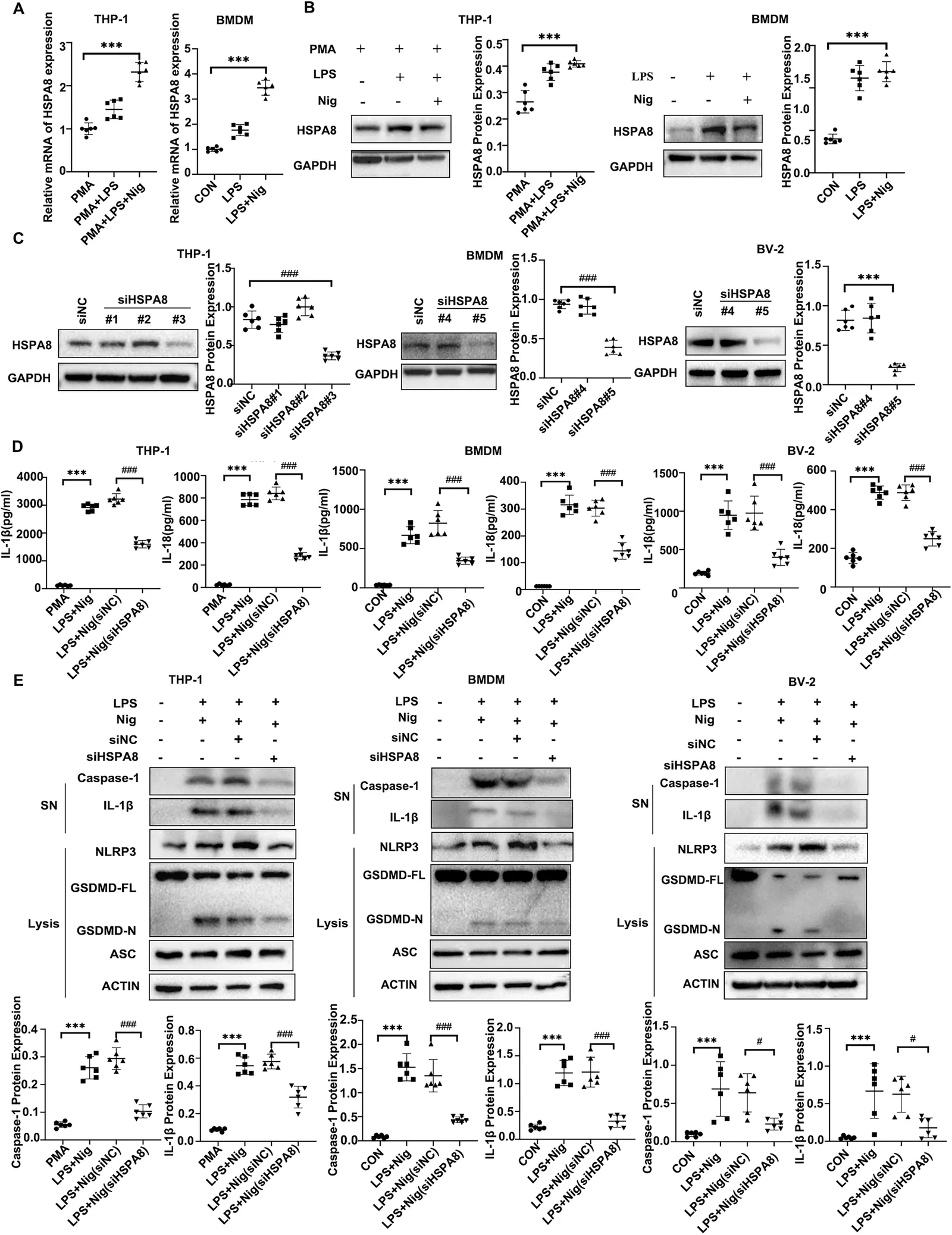
HSPA8 knockdown attenuates the activation of NLRP3 inflammasome in vitro. NLRP3 inflammasome activation was induced by LPS and nigericin. HSPA8 expression was assessed by qPCR (**A**) and Western blot (**B**) (*n* = 6). **C** Knockdown efficiency of human or mouse HSPA8 siRNA (siHSPA8) versus non-targeting control siRNA (siNC) in PMA-differentiated THP-1 cells, BMDMs, and BV-2 microglial cells, confirmed by Western blot after 48 h (*n* = 6). **D**, **E** Cells transfected with siHSPA8 or siNC were primed with LPS and subjected to stimulation with nigericin. Secreted IL-1β and IL-18 were measured by ELISA (D), while NLRP3 inflammasome-associated proteins were analyzed by Western blot (**E**) (*n* = 6). Data are mean ± SD. ^***^P < 0.001, vs. PMA. ^***^P < 0.001, vs. CON. ^*#*^*P* < 0.05, ^*##*^*P* < 0.01, ^*###*^*P* < 0.001, vs. LPS^*+*^ Nig (siNC). The data were analyzed using one-way analysis of variance, followed by Tukey’s multiple comparison test.

To dissect the mechanism underlying HSPA8-mediated regulation of the NLRP3 inflammasome, we turned to cellular models. We activated the NLRP3 inflammasome in PMA-differentiated THP-1 cells and BMDMs using LPS (priming) followed by nigericin (activation). Upon inflammasome activation, HSPA8 expression was significantly induced at both the mRNA and protein levels in both cell types compared to their respective unstimulated controls (Fig. 4A, B).

We next designed and transfected species-specific siRNAs targeting HSPA8 (siHSPA8) into THP-1, BMDMs, and BV-2 cells. After screening for efficacy, one optimal siRNA for each species was selected for subsequent experiments (siRNA3 for human, siRNA5 for murine) (Fig. 4C). Functional experiments showed that activation of the NLRP3 inflammasome in THP-1 cells and BMDMs induced pyroptosis (Supplementary Fig. 2) and secretion of IL-1β and IL-18 (Fig. 4D). Notably, these responses were markedly reduced following HSPA8 knockdown, suggesting that HSPA8 is functionally associated with inflammasome-mediated cytokine release and pyroptosis. Western blot analysis further corroborated these findings, with HSPA8 knockdown leading to reduced levels of caspase-1 and IL-1β in the supernatants, as well as the pyroptosis executioner protein GSDMD-N in cell lysates (Fig.4E). To assess the relevance of these findings in a CNS-associated innate immune cell context, we repeated the above experimental procedure using mouse BV2 microglia. Consistent with the results in THP-1 and BMDMs, HSPA8 knockdown attenuated NLRP3 inflammasome-related responses in BV2 cells (Fig. 4D, E), supporting a reproducible effect of HSPA8 knockdown in a microglial cell context. We further performed a rescue experiment in THP‑1 cells. Transfection with an siRNA‑resistant HSPA8 plasmid substantially restored HSPA8 protein levels and partially restored NLRP3 inflammasome activation, including IL‑1β release and caspase‑1 cleavage, supporting that the inhibitory effect of siHSPA8 was primarily target-specific (Supplementary Fig. 3).

### HSPA8 knockdown also attenuates AIM2 and NLRC4 inflammasome activation

**Fig. 5:**
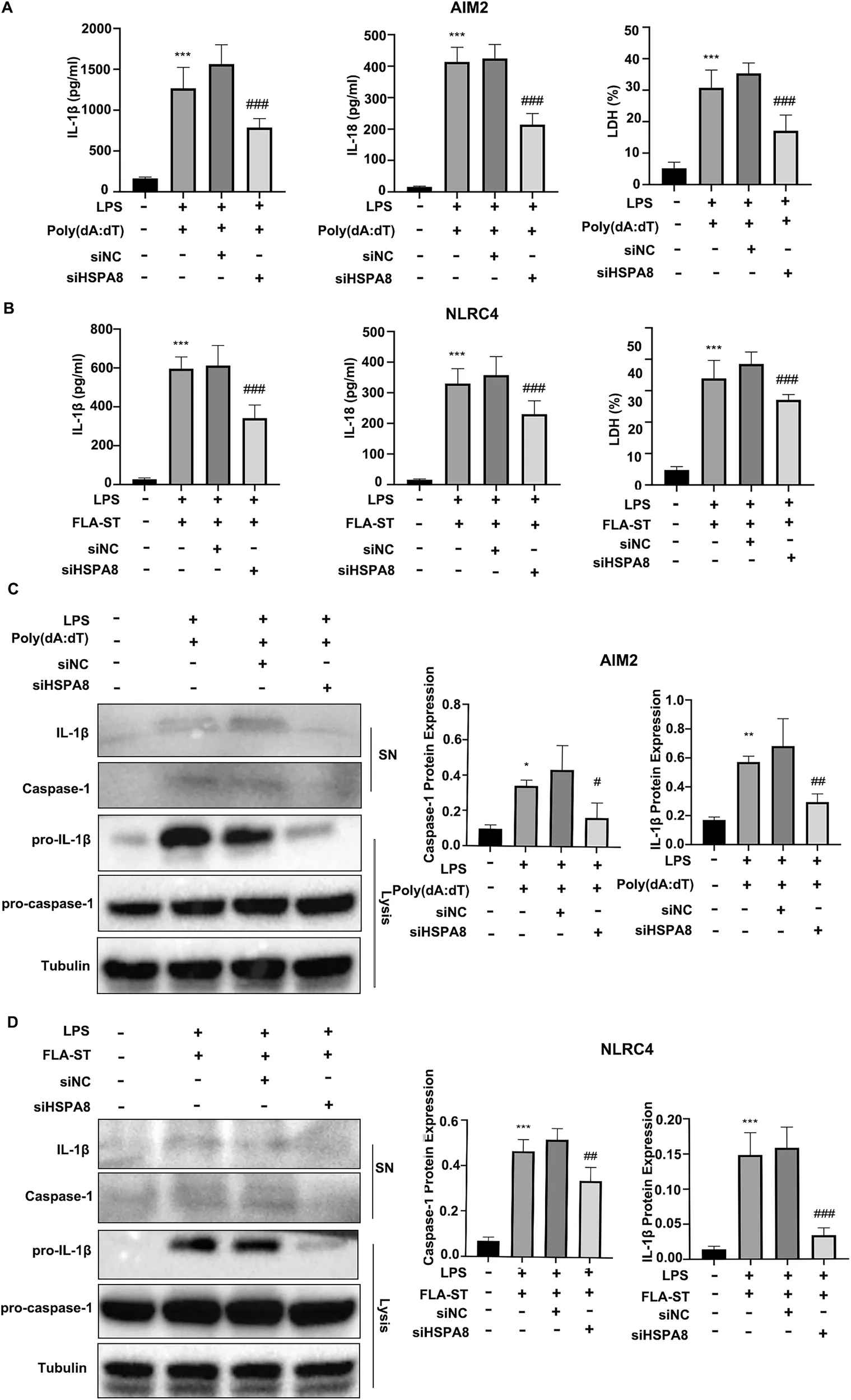
HSPA8 knockdown attenuates AIM2 and NLRC4 inflammasome activation. PMA-differentiated THP-1 cells were transfected with siNC or siHSPA8, then primed with LPS. Cells were subsequently treated with poly(dA:dT) to induce the AIM2 inflammasome or with FLA-ST to induce the NLRC4 inflammasome. **A**, **B** Secretion of IL-1β and IL-18 was measured by ELISA following AIM2 (**A**, left) or NLRC4 (**B**, left) activation (*n* = 6). LDH release was measured to assess pyroptotic cell death after AIM2 (**A**, right) or NLRC4 (**B**, right) inflammasome activation (*n* = 6). Western blot analysis of supernatants for mature IL-1β and cleaved caspase-1, and cell lysates for pro-IL-1β and pro-caspase-1, following AIM2 (**C**) or NLRC4 (**D**) activation (*n* = 6). Data are mean ± SD. ^***^P < 0.001, vs. CON. ^*##*^*P* < 0.01, ^*###*^*P* < 0.001, vs. LPS ^*+*^ poly(dA:dT) (siNC). ^*##*^*P* < 0.01, ^*###*^*P* < 0.001, vs. LPS ^*+*^ FLA-ST (siNC). The data were analyzed using one-way analysis of variance, followed by Tukey’s multiple comparison test.

We next examined whether the effect of HSPA8 knockdown could also be observed in other inflammasome activation models. In PMA-differentiated THP-1 cells, activation of the AIM2 inflammasome (via poly(dA:dT) transfection) or the NLRC4 inflammasome (via FLA-ST transfection) robustly induced the secretion of IL-1β and IL-18 (Fig. 5A, B). HSPA8 knockdown attenuated cytokine release induced by both stimuli (Fig. 5A, B). Consistent with this, Western blot analysis showed that HSPA8 knockdown reduced the processing of caspase-1 and IL-1β upon AIM2 or NLRC4 activation (Fig. 5C, D). Additionally, HSPA8 knockdown reduced cell pyroptosis induced by AIM2 and NLRC4 activation (Fig. 5A, B). Collectively, these data indicate that HSPA8 knockdown also attenuated the activation of AIM2 and NLRC4 inflammasomes under the present experimental conditions.

### HSPA8 knockdown attenuates NF-κB-dependent priming of the NLRP3 inflammasome

**Fig. 6:**
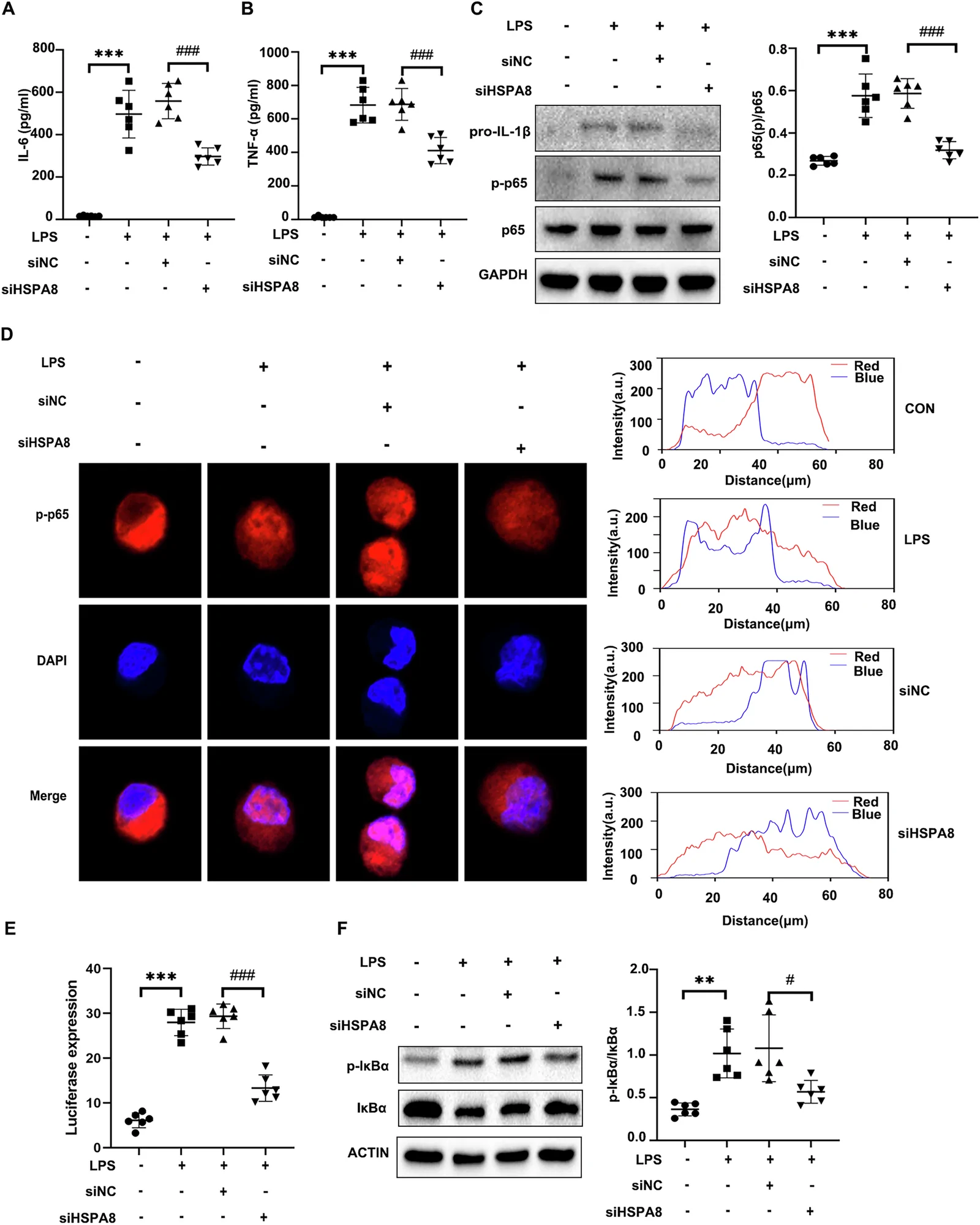
HSPA8 knockdown is associated with impaired NLRP3 inflammasome priming and reduced NF-κB signaling activity. PMA-differentiated THP-1 cells were transfected with siNC or siHSPA8, followed by LPS stimulation to activate NF-κB. Secretion of the NF-κB-dependent cytokines IL-6 (**A**) and TNF-α (**B**) was measured by ELISA (*n* = 6). **C** Western blot analysis of pro-IL-β, phospho-NF-κB p65 (p-p65) and total p65 (*n* = 6). **D** Immunofluorescence analysis of phospho-p65 nuclear translocation. Nuclei were counterstained with DAPI (*n* = 6). **E** NF-κB transcriptional activity was assessed using a luciferase reporter assay (*n* = 6). **F** Western blot analysis of p-IκBα and total IκBα (*n* = 6). Data are mean ± SD. ^**^P < 0.01, ^***^P < 0.001 vs. PMA. ^*#*^*P* < 0.05, ^*###*^*P* < 0.001 vs. LPS (siNC). ns, statistically insignificant. The data were analyzed using one-way analysis of variance, followed by Tukey’s multiple comparison test.

Inflammasome NLRP3 activation typically follows a “two-step” model (priming → assembly): Priming is mainly mediated by TLR/cytokines, and the transcription of NLRP3 and pro-IL-1β is upregulated via NF-κB, thereby providing a foundation for subsequent assembly and activation “licensing.” To further elucidate how HSPA8 influences NLRP3 inflammasome activation, we evaluated whether it regulates the “priming” stage. LPS significantly induced the release of inflammatory cytokines such as IL-6 and TNF-α in THP-1 cells, but this effect was reduced following HSPA8 knockdown (Fig. 6A, B). Moreover, Western blot showed that HSPA8 knockdown decreased LPS-induced pro-IL-1β expression (Fig. 6C). Additionally, HSPA8 knockdown markedly decreased the phosphorylation of NF-κB p65 protein and inhibited its nuclear translocation in LPS-stimulated THP-1 cells (Fig. 6C, D). To further elucidate the functional regulation of the NF-κB pathway by HSPA8, we performed an NF-κB luciferase reporter assay. As shown in Fig. 6E, LPS stimulation significantly increased NF-κB reporter activity, whereas this response was reduced in HSPA8 knockdown cells, indicating that HSPA8 deficiency reduces NF-κB–dependent transcriptional responses. To investigate the upstream molecular events, we examined the phosphorylation and degradation of IκBα. As shown in Fig. 6F, HSPA8 knockdown reduced LPS-induced IκBα phosphorylation, suggesting a potential involvement of HSPA8 in upstream NF-κB signaling. Collectively, these results indicate that HSPA8 knockdown is associated with impaired NF-κB activation and reduced NLRP3/pro-IL-1β expression, supporting a functional link between HSPA8 and NF-κB-dependent priming.

### HSPA8 interacts with the ASC protein

**Fig. 7:**
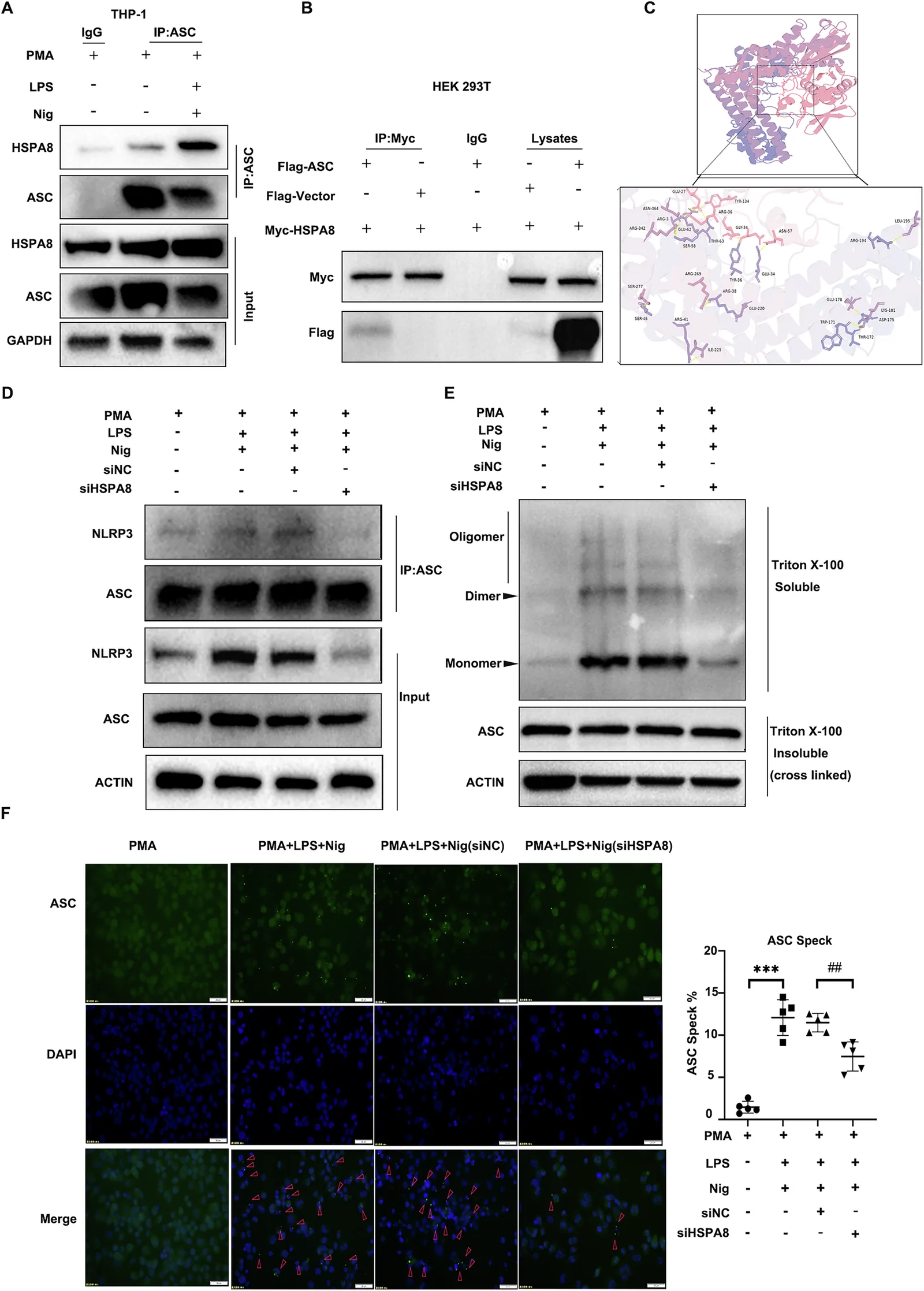
HSPA8 knockdown impairs NLRP3-ASC interaction, ASC oligomerization, and ASC speck formation during inflammasome assembly. **A** For the endogenous interaction detection, Co-IP was conducted in THP-1-derived macrophages after NLRP3 inflammasome activation. Immunoprecipitation of cell lysates was carried out using an anti-ASC antibody or control IgG, with subsequent immunoblotting targeting HSPA8 (*n* = 6). **B** For the ectopic expression interaction, HEK293T cells were co-transfected with plasmids encoding Flag-tagged ASC and Myc-tagged HSPA8. Co-IP assays were conducted on cell lysates with anti-Myc antibody, with subsequent immunoblotting performed using the anti-Flag antibody (*n* = 6). **C** Structural prediction: Molecular docking analysis predicts a potential binding interface between HSPA8 and ASC. For functional assays (**D**–**F**), PMA-differentiated THP-1 cells (transfected with siNC or siHSPA8) were primed with LPS and stimulated with nigericin to activate the NLRP3 inflammasome. **D** ASC oligomerization was assessed by chemical cross-linking with DSS, followed by Western blot analysis of cross-linked high-molecular-weight complexes under non-reducing conditions (*n* = 6). **E** To analyze the NLRP3-ASC interaction, Co-IP was carried out with an anti-ASC antibody, and NLRP3 was subsequently detected by immunoblotting (*n* = 6). **F** Formation of ASC specks was visualized by immunofluorescence. Arrows indicate specks. Scale bar, 20 μm (*n* = 6). Data are mean ± SD. ^***^P < 0.001, vs. PMA. ^*##*^*P* < 0.01 vs. LPS+Nig (siNC). The data were analyzed using one-way analysis of variance, followed by Tukey’s multiple comparison test.

Given the broad inhibitory effect of HSPA8 knockdown on multiple inflammasomes (NLRP3, AIM2, NLRC4) and the central role of ASC as a common adaptor, we examined whether HSPA8 interacts with ASC. Endogenous Co-IP analysis demonstrated that HSPA8 associates with ASC, and this interaction was enhanced upon inflammasome activation (Fig. 7A). We further examined this interaction using exogenous Co-IP. In HEK293T cells co-expressing Myc-tagged HSPA8 and Flag-tagged ASC, ASC was reciprocally co-immunoprecipitated with HSPA8, consistent with an interaction between the two proteins (Fig. 7B). To gain structural insights, we performed molecular docking between the crystal structures of ASC (PDB ID: 2KN6) and HSPA8 (PDB ID: 3LDQ) using the ZDOCK server. Docking predictions suggested that the polar amino acids within HSPA8 (GLU27, TYR134, ASN57, etc.) may interact with residues in ASC (GLU62, TRP171, ARG38, etc.) via multiple hydrogen bonds and electrostatic interactions (Fig. 7C). Collectively, these data support a physical association between HSPA8 and ASC, and provide a hypothesis-generating binding model that warrants further investigation.

### HSPA8 knockdown impairs NLRP3–ASC interaction, ASC oligomerization, and ASC speck formation during inflammasome assembly

The NLRP3 protein enlists the adaptor protein ASC through its PYD domain, inducing the oligomerization of ASC molecules and formation of the typical intracellular “speck” structure. This is a key step in the assembly and activation of the NLRP3 inflammasome, the subsequent activation of downstream caspase-1, and the release of inflammatory factors, including IL-1β. Therefore, the influence of HSPA8 on this process was further investigated systematically. As shown in Fig. 7D, LPS and nigericin enhanced the interaction between NLRP3 and ASC in THP-1 cells, whereas this interaction was attenuated following HSPA8 knockdown (Fig. 7D). DSS cross-linking experiments showed that HSPA8 knockdown significantly reduced the formation of ASC oligomers (Fig. 7E). Additionally, after induction of the activation of NLRP3 in THP-1 cells by LPS and nigericin, ASC aggregated to form speck (Fig.7F). However, the number of these specks was reduced in the HSPA8 knockdown group (Fig. 7F). These findings indicate that HSPA8 is functionally associated with NLRP3–ASC interaction, ASC oligomerization, and ASC speck formation. HSPA8 knockdown impaired these assembly-related processes, thereby attenuating the activation of NLRP3 inflammasome.

## Discussion

**Fig. 8:**
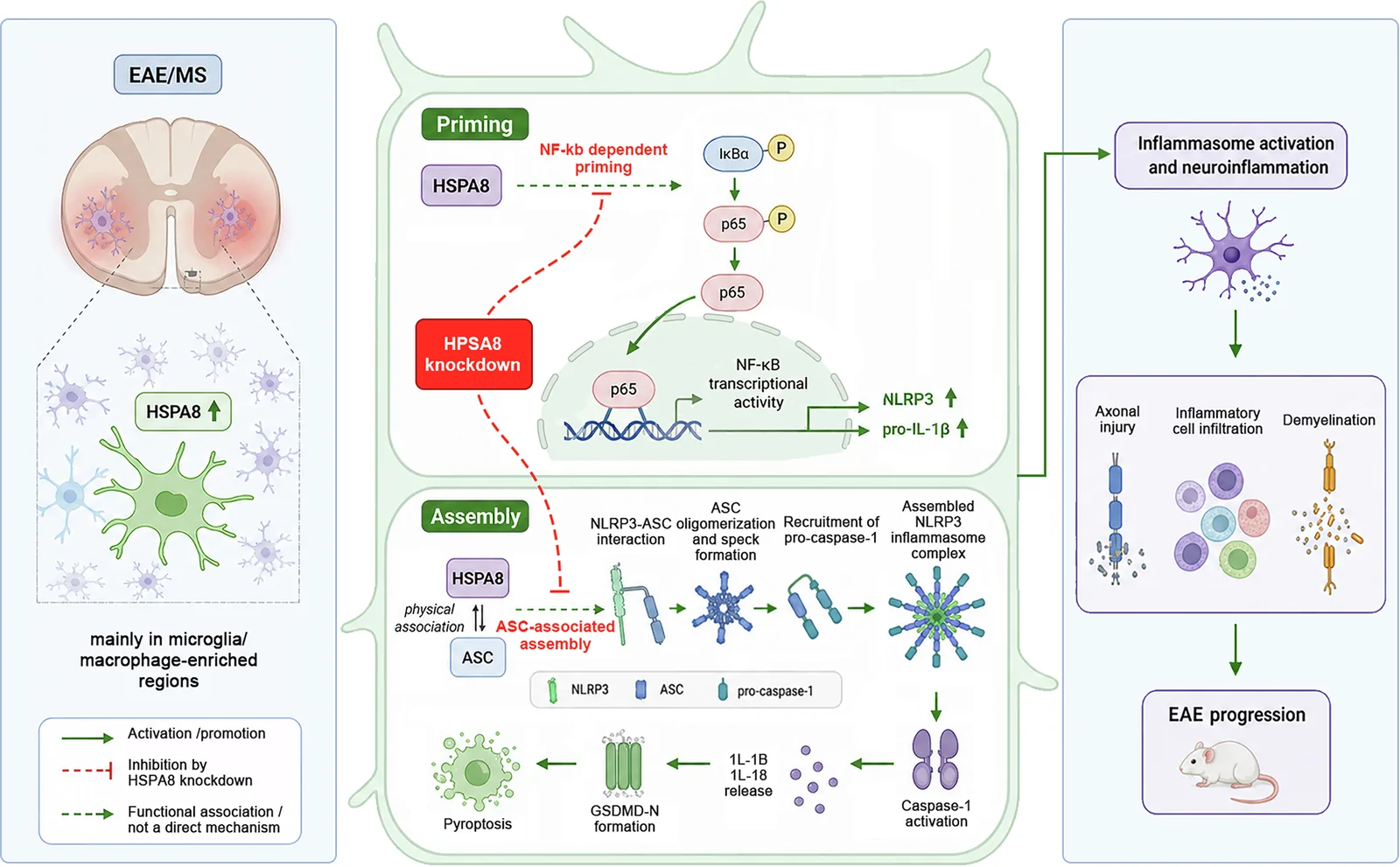
Schematic diagram of the role of HSPA8 in EAE mice. Solid lines indicate experimentally supported associations, whereas dashed lines indicate hypothetical mechanisms requiring further validation. HSPA8 is proposed to be functionally associated with both NF-κB-dependent priming and ASC-associated assembly of the NLRP3 inflammasome in EAE. HSPA8 knockdown impaired the priming stage, as reflected by reduced p65 phosphorylation, nuclear translocation, NF-κB transcriptional activity, and pro-IL-1β expression. It also impaired assembly-related events, including NLRP3–ASC interaction, ASC oligomerization, and ASC speck formation. These changes were associated with reduced inflammasome activation and decreased IL-1β/IL-18 release.

MS is an autoimmune disorder that causes severe neurological impairment, for which the underlying molecular mechanisms remain only partially understood, and effective targeted therapies are still lacking. Our findings suggest that HSPA8 is markedly upregulated in EAE mice, and HSPA8 knockdown significantly alleviates the severity of the disease. Notably, HSPA8 upregulation in EAE spinal cords was more prominently associated with Iba1-positive microglia/macrophage-enriched regions than with GFAP- or NeuN-positive regions, providing cellular-context support for linking HSPA8 to CNS innate immune activation. Mechanistically, our data suggest that HSPA8 is functionally associated with two key stages of NLRP3 inflammasome activation: NF-κB-dependent “priming” and ASC-associated “assembly”. HSPA8 knockdown was associated with reduced NF-κB signaling activity, impaired NLRP3–ASC interaction, and decreased ASC oligomerization and speck formation, ultimately attenuating neuroinflammatory responses in EAE (Fig. 8). These findings support a pro-inflammatory role of HSPA8 in EAE pathogenesis and highlight HSPA8 as a candidate target with potential therapeutic relevance for MS.

As a constitutively expressed member of the HSP70 family, HSPA8 plays an indispensable role in maintaining protein homeostasis through protein folding, degradation, and chaperone-mediated autophagy. It also plays multiple roles in cellular stress responses and immune regulation [20, 21]. However, the function of HSPA8 appears to be context-dependent. Different HSP70 family members exert diverse, sometimes opposing, effects in neurological diseases. For instance, overexpression of HSP70 and HSP40 in a PD model promotes the clearance of high-molecular-weight α-synuclein, providing neuroprotection [27, 28]. In contrast, HSPA8 expression can increase significantly in animal models of brain ischemia, and its downregulation is helpful for the recovery of neurological functions [29–31]. Together, these studies indicate that HSPA8 is not uniformly protective or detrimental, but may exert different biological effects depending on disease context and inflammatory status. In contrast to its reported neuroprotective roles in ischemic settings, our data suggest that HSPA8 may act as a disease-promoting factor in autoimmune neuroinflammation, likely through its association with inflammasome activation. This functional divergence aligns with the emerging view that chaperone activity is dynamically shaped by the inflammatory milieu.

In this study, HSPA8 knockdown significantly alleviated EAE and neuroinflammatory responses in model mice. Notably, IL-1β and IL-18, two canonical inflammasome-dependent cytokines, were more prominently reduced after HSPA8 knockdown than other inflammatory cytokines. Together with the preferential association of HSPA8 upregulation with Iba1-positive microglia/macrophage-enriched regions, this cytokine profile provided cellular and inflammatory support for linking HSPA8 to inflammasome-mediated neuroinflammation in EAE. Given that NLRP3 inflammasome components are elevated in MS patient serum and cerebrospinal fluid [8‑12], these findings further support our focus on aberrant inflammasome activation.

Among inflammasomes, NLRP3 is the most thoroughly characterized one. It is known to exacerbate MS pathology through multiple mechanisms, including amplifying the neuroinflammatory response, disrupting the blood-brain barrier, and inhibiting remyelination [8, 15]. Our findings are consistent with studies showing that pharmacological inhibition of NLRP3 with MCC950 [15] or OLT1177 [16] ameliorates EAE, and further suggest that HSPA8 may represent an endogenous factor functionally linked to NLRP3 inflammasome activation in this disease context. Notably, the effect of HSPA8 may not be restricted to the NLRP3 pathway. The downregulation of HSPA8 also has an inhibitory effect on the activation of the AIM2 and NLRC4 inflammasomes to a certain extent. However, the current data on AIM2 and NLRC4 remain descriptive and preliminary, and the specific molecular mechanisms—including whether HSPA8 directly interacts with AIM2 or NLRC4 sensors, or acts entirely through ASC—have not been clarified. Because ASC serves as a common adaptor for multiple inflammasomes, this cross-inflammasome effect may introduce additional complexity in interpreting the in vivo phenotype. This cross‑inflammasome inhibitory pattern is reminiscent of the effects observed with ASC‑targeting strategies, such as lonidamine [19], raising the possibility that HSPA8 may influence inflammasome signaling at the level of the shared adaptor ASC rather than through sensor-specific regulation alone. Therefore, the detailed regulatory mechanisms for these inflammasome subtypes require further investigation. Overall, these findings support a pathogenic association between HSPA8 and inflammasome-mediated neuroinflammation in EAE, while also suggesting that HSPA8 may influence broader inflammasome signaling networks under specific experimental conditions.

Activation of the NLRP3 inflammasome is a tightly regulated two-phase process, consisting of the “priming” and “assembly” stages. Prior research has discovered that various drugs or interventions can suppress the NF-κB pathway, thereby alleviating the abnormal activation of inflammasomes such as NLRP3. Therefore, these agents are effective in alleviating pathological conditions such as bronchial asthma, heart failure, diabetes, gouty arthritis, and PD [32–36]. However, conclusions regarding the regulatory role of HSPA8 in the NF-κB pathway vary in the literature. Some studies have reported that inhibiting HSPA8 can suppress the inflammatory response by blocking the NF-κB pathway, thereby protecting spinal cord astrocytes from oxygen-glucose deprivation/re-oxygenation–induced ischemia-reperfusion injury [23]. However, other studies have shown that upregulating HSPA8 can inhibit the NF-κB signaling pathway and STAT3, thereby suppressing the neuroinflammatory response in PD [37]. The reasons for these functional differences may be connected to factors such as the type and intensity of stimulation, cell type, and timing. In the present study, HSPA8 knockdown was associated with reduced NF-κB p65 phosphorylation, nuclear translocation, IκBα phosphorylation, and NF-κB-dependent transcriptional activity. Consistently, HSPA8 knockdown in EAE mice reduced the expression of priming-related inflammasome proteins, including NLRP3 and pro-IL-1β, in spinal cord tissues. These findings support a functional link between HSPA8 and NF-κB-dependent priming of the NLRP3 inflammasome in EAE. However, the precise molecular target of HSPA8 within the IKK–IκBα–p65 cascade has not been definitively identified.

During inflammasome assembly, ASC serves as a critical scaffolding platform, linking NLRP3 to caspase-1 to mediate inflammasome assembly. Moreover, ASC oligomers coalesce into distinct ASC specks, which amplify the inflammatory signal by enhancing caspase-1 activity [38, 39]. Our results demonstrate that HSPA8 knockdown significantly inhibited NLRP3 inflammasome activation and also diminished AIM2 and NLRC4 inflammasome activity. Given that ASC is a common adaptor for these inflammasomes, these findings raised the possibility that HSPA8 may influence inflammasome assembly at the level of ASC. Consistent with this idea, Co-IP assays showed that HSPA8 associates with ASC, and this association was enhanced during the activation of inflammasomes. Results from molecular docking predicted that multiple sites between HSPA8 and ASC may form stable binding conformations via hydrogen bonding and electrostatic interactions, providing a working hypothesis for future structural investigation. Importantly, HSPA8 knockdown weakened the NLRP3-ASC interaction and significantly inhibited subsequent ASC oligomerization and speck formation. This chaperone‑assisted assembly mechanism parallels the role of HSPA8 in other multiprotein complex formations, such as its interaction with NLRC5 in cardiac remodeling [22], and extends the functional repertoire of HSP70 family members beyond protein quality control to include functional participation in innate immune signaling platforms.

Our study has several limitations. First, although co-staining suggested that HSPA8 upregulation in EAE spinal cords was preferentially associated with Iba1-positive microglia/macrophage-enriched regions, these data do not establish definitive cell-type-specific localization. Moreover, the AAV-mediated knockdown used in vivo was not cell-type specific, thus, the observed effects cannot be attributed exclusively to microglia/macrophages and may reflect contributions from multiple CNS-resident and infiltrating immune cell populations. Second, the precise binding interfaces and structural basis of the HSPA8–ASC interaction remain undefined and require structural and mutational validation. Third, the strong in vitro inflammasome stimuli used here may not fully recapitulate chronic neuroinflammatory conditions in EAE/MS. Fourth, AAV-shHSPA8 was administered in a preventive setting; whether HSPA8 inhibition after disease onset confers therapeutic benefit remains to be determined. Fifth, although key findings were validated in BV2 microglia, further confirmation in primary microglia or more physiologically relevant systems is warranted. Finally, this study is mainly based on the EAE animal model and in vitro cell systems, and lacks validation using human MS lesion tissues or large-scale genetic data.

In conclusion, this study elucidates the critical role of HSPA8 in EAE pathogenesis. Mechanistically, HSPA8 appears to be functionally associated with both NF-κB-dependent priming and ASC-dependent assembly of the NLRP3 inflammasome. These findings identify HSPA8 as a pivotal regulatory node linking proteostasis to inflammasome amplification, presenting HSPA8 inhibition as a candidate strategy for MS and other inflammasome-driven neuroimmune disorders.

## Methods

### Main antibodies and reagents

Antibodies for Western blot/Immunoprecipitation/Immunofluorescence: Anti-caspase-1 (ab207802), Anti-pro-caspase-1 (ab179515), Anti-HSC70/HSPA8 (ab51052), and Anti-IL-1β (ab254360) were from Abcam (Cambridge, UK). Anti-GSDMD (39754S) was from Cell Signaling Technology (Danvers, USA). Anti-NLRP3 (AG-20B-0014) and Anti-ASC (AG-25B-0006) were from Adipogen (San Diego, CA, USA). Anti-GAPDH (GB15004) and Anti-β-actin (GB15003) were from Servicebio (Wuhan, China). Anti-phospho-nuclear factor kappa-B (NF-κB) p65 (sc-135769) was from Santa Cruz Biotechnology (Dallas, TX, USA). NF-κB p65 Recombinant Rabbit mAb (S-475-56)(S0B0549) was purchased from Starter (Univ, Shanghai, China). Anti-β-tubulin (AF7011) was from Affinity Biosciences (Cincinnati, OH, USA). c-Myc Tag (9E10) (MA1980) and DYKDDDDK (FLAG) Tag (FG4R) (MA1-91878) were from Thermo Fisher Scientific (Waltham, MA, USA). Antibodies for Immunohistochemistry/Immunofluorescence: Anti-myelin basic protein (MBP) (GB12226), Anti-Iba1 (GB12105), anti-GFAP (GB11100), and anti-NeuN (GB11138) were from Servicebio. Anti-neurofilament (NF) antibody (sc-51683) was from Santa Cruz. Secondary Antibodies & Detection Reagents: HRP-conjugated Anti-Rabbit IgG (AK-111-035-003) and HRP-conjugated Anti-Mouse IgG (AK-115-035-003) were from ACRED Biological Testing Lab (Shanghai, China). FITC-conjugated Goat Anti-Rabbit IgG (A0562), Cy3-conjugated Goat Anti-Mouse IgG (A0521), and Human IgG Isotype Control (A7001) were from Beyotime (Shanghai, China).

Inflammasome Activators & Reagents: Lipopolysaccharide (LPS, E. coli O111:B4) (L2630) and Nigericin (HY-100381) were from nigericin MedChemExpress (Monmouth Junction, NJ, USA). FLA-ST (Flagellin from S. typhimurium) (tlrl-stfla (5982-46-01)) and poly(dA:dT) (tlrl-patn (6249-45-03)) were from InvivoGen (San Diego, CA, USA). Kits & Special Reagents: NF-κB Activation-Nuclear Translocation Assay Kit (SN368) was from Beyotime. Pierce™ Co-Immunoprecipitation Kit and Dithiobis (succinimidyl propionate) (DSS) were from Thermo Fisher Scientific. DAPI Staining Solution (AR1176) was from Boster Biological Technology (Pleasanton, CA, USA). Cell Culture & Transfection Reagents: Advanced DNA RNA Transfection Reagent (AD600150) was from Zeta Life (Menlo Park, CA, USA). Lipofectamine 2000 (11668-019) and Lipofectamine RNAi MAX (13778) were from Invitrogen (Carlsbad, CA, USA). PMA (P8139) was from Sigma-Aldrich (St. Louis, MO, USA). Plasmids: pCMV-FLAG-HSPA8 (Human) and empty vector control were from Shanghai Jima Pharmaceutical (Shanghai, China). pCMV-Myc-ASC (Human) and empty vector control were from Zhengzhou Luer Biotechnology (Zhengzhou, China).

### Mice

Six- to eight-week-old female C57BL/6 mice, sourced from Beijing Sibeifu Laboratory Animal Technology Co., Ltd. (Beijing, China), were used in this study. Animals were housed in an environmentally controlled facility with a 12 h light/12 h dark cycle, specific temperature and humidity, and ad libitum access to food and water. All experimental procedures involving animals were reviewed and approved by the Animal Experiment Ethics Committee of the First Affiliated Hospital of Henan University of Science and Technology.

### Experimental design

For determining the impact of HSPA8 knockdown on EAE, 72 mice were randomly assigned to 4 groups. No statistical differences in age or weight were observed among the groups. The groups were as follows: (1) Sham-control group (CON) (*n* = 18): mice were injected with complete Freund’s adjuvant (CFA) emulsion without antigen: Only complete Freund’s adjuvant was injected as a healthy control; (2) EAE group (*n* = 18): the MOG_35-55_/CFA emulsion was injected to induce the EAE model; (3) EAE + AAV-CON group (*n* = 18): the control group Adeno-Associated Virus (AAV) (AAV-CON) was injected intrathecally, and EAE modeling was performed 2 weeks later; (4) EAE + AAV-shHSPA8 group (*n* = 18): the adenovirus targeting the silencing of HSPA8 (AAV-shHSPA8) was injected intrathecally, and EAE modeling was performed 2 weeks later. The adenovirus was provided by Genechem (Shanghai, China).

### Induction and evaluation of EAE

Female C57BL/6 mice aged 6-8 weeks were subcutaneously immunized on the dorsum with 100 μg MOG_35-55_ peptide (Qiangyao Biotechnology) per mouse, emulsified in Complete Freund’s adjuvant (CFA; 5 mg/mL) containing *Mycobacterium tuberculosis*. On the day of vaccination and 48 h later, each mouse received an intraperitoneal injection of 200 ng of pertussis toxin (Listlab, LISTBio#181). The day of immunization was day 0. The animals were monitored and scored every day until they were euthanized. The clinical score assessment is as follows: 0 = no clinical symptoms, 1 = tail paralysis, 2 = gait imbalance, mild paralysis of hind limbs, 3 = bilateral hind limb paralysis, 4 = forelimb paralysis, 5 = dying or death. If the score is between two points, 0.5 points is used. The evaluators were unaware of the specific grouping of the experimental animals.

### AAV construction and intrathecal injection

AAV9 carrying shHSPA8 (targeting mouse HSPA8, GenBank NM_031165) or scramble control was packaged (Genechem) under a CMV promoter (titer 1 × 10¹² vg/mL). Intrathecal injection at L5-L6: 5 µL (5 × 10⁹ vg) at 5 µL/min, needle left for 2 min. Mice recovered for 2 weeks before EAE induction. Knockdown efficiency was confirmed by qPCR/Western blot.

### Cell culture

Bone marrow-derived macrophages (BMDMs) of primary origin were obtained from 6- to 8-week-old C57BL/6 mice and subjected to 7 days of differentiation in DMEM containing 10% FBS (Gibco, A5669701), 1 mM sodium pyruvate, 1% penicillin/streptomycin (P/S), 2 mM L-glutamine, and 50 ng/mL M-CSF (macrophage colony-stimulating factor; PEPROTECH, 316-02). HEK293T cells (Procell), BV-2 cells (Yuanjing Biotechnology) and THP-1 cells (Yuanjing Biotechnology) were cultured in DMEM with 10% FBS and 1% P/S and in THP-1-specific medium (Procell), respectively.

### Inflammasome activation

5 × 10^5^ cells/mL THP-1 were inoculated into 6-well plates containing 100 nM PMA medium and cultured overnight. To induce NLRP3 inflammasome activation, cells were incubated with LPS at 50 ng/mL for 3 h, prior to being stimulated with nigericin (3 μM) for 30 minutes. To induce absent in melanoma 2 (AIM2) inflammasome activation, cells were treated with LPS (500 ng/mL) for 4 h, prior to being transfected with poly(dA:dT) using Lipofectamine 2000 following the manufacturer’s specifications for 6 h. To induce NLR family CARD domain containing 4 (NLRC4) inflammasome activation, cells were first treated with LPS (500 ng/mL) for 4 h to initiate, and then transfected with FLA-ST using Lipofectamine 2000 for 6 h.

5 × 10^5^ cells/mL of BMDM were inoculated in 6-well plates and incubated overnight. The next morning, the existing culture medium was replaced with fresh, sterile medium. To induce the activation of NLRP3 inflammasome, the cells were stimulated with LPS (500 ng/mL) for 3 h and with nigericin (10 μM) for 1 h.

### siRNA interference

THP-1 or BMDM cells were seeded in 6-well plates at a density of 3 × 10^5^ cells/mL one day in advance. The culture medium was replaced with Opti-MEM. RNA interference was performed using Advanced DNA RNA Transfection Reagent or Lipofectamine™ RNAi MAX following the manufacturer’s protocol. The siRNA sequences were chemically synthesized by GenePharma (Shanghai, China) and are as follows: transfection of PMA-induced THP-1 cells with siHSPA8#1 (5′-CUUGAGUCCUAUGCCUUCATT-3′), siHSPA8#2 (5′-AAGCUGUACCAGAGUGCAGTT-3′), and siHSPA8#3 (5′-CUGGACAAGUGUAAUGAAATT-3′). Transfection of BMDM cells with siHSPA8#4 (5′-GATCGATTCTCTCTATGAA-3′) and siHSPA8#5 (5′-GTCTCAATGTACTTAGAAT-3′).

### siRNA rescue

THP-1 cells were co-transfected with siNC + empty vector (control), siHSPA8 + empty vector (knockdown), or siHSPA8 + siRNA-resistant HSPA8 plasmid (HSPA8-rescue, GenePharma). Rescue plasmid has synonymous mutations in the siRNA target site. Cells were harvested 48–72 h post-transfection.

### Enzyme-linked immunosorbent assay (ELISA)

According to the manufacturer’s instructions, analyze the cell culture supernatants and serum for Mouse IL-1β ELISA Kit (Elabscience, E-EL-M0037), Human IL-1β ELISA Kit (Boster, EK0392), Mouse IL-18 ELISA Kit (Elabscience, E-EL-M0730), Human IL-18 ELISA Kit (Boster, EK0864), Mouse tumor necrosis factor (TNF)-α ELISA Kit (Elabscience, E-EL-M3063), and Human TNF-α ELISA Kit (Boster, EK0525).

### Western blot analysis

Cells were lysed using strong RIPA lysis buffer (Biosharp, BL504A). Then, total protein concentration was thereafter quantified using the Bicinchoninic Acid Assay Kit (Biosharp, BL521B). 20–40 μg of protein from each sample was loaded onto 10% or 12.5% SDS-PAGE gels for separation and transferred to polyvinylidene fluoride membranes (Millipore, IPVH00010). After being blocked with 5% skim milk blocking solution for 1 h at room temperature, the membrane was subjected to overnight incubation at 4 °C with the primary antibody diluted as per the manufacturer’s instructions. Subsequently, the membrane was rinsed with TBST (Tris-buffered saline containing Tween 20) and subjected to incubation with HRP-labeled goat anti-rabbit or anti-mouse secondary antibodies for 1 h at room temperature. Visualization was performed using a chemiluminescence kit (Epizyme, SQ201).

### Cell apoptosis determination

The procedure was performed based on the instructions of the Lactate Dehydrogenase (LDH) Assay Kit (Beyotime, C0016) to evaluate LDH release.

### Luciferase reporter assay

To assess the effect of HSPA8 knockdown on NF-κB transcriptional activity, PMA-differentiated THP-1 cells were co-transfected with pNF-κB-TA-luc reporter (Beyotime, D2207) and pRL-TK (Renilla) (Beyotime, D2760) using Lipofectamine 3000. After 24 h, cells were transfected with siNC or siHSPA8 for another 48 h, then stimulated with LPS (1 µg/mL) for 30 min. Luciferase activity was measured using a dual luciferase reporter assay kit (Beyotime, RG027). NF-κB activity was expressed as the firefly/Renilla luciferase ratio.

### Immunoprecipitation experiment (Co-IP)

For the determination of endogenous interactions, THP-1 cells were seeded in 10 cm dishes and stimulated. The cells were subsequently harvested and lysed in a cell lysis buffer supplemented with protease inhibitors. Perform immunoprecipitation and elution as per the manufacturer’s instructions, and evaluate by Western blot.

For the determination of exogenous interactions, HEK293T cells were seeded in a 10 cm culture dish and transfected with the plasmid using Lipofectamine 2000. After an incubation period of 72 h, the cells were collected and subjected to lysis with a cell lysis buffer supplemented with protease inhibitors. The extracts were immunoprecipitated using specific antibodies and immunoprecipitation kit reagents, and evaluated by Western blot.

### Real-time quantitative polymerase chain reaction (qPCR)

Total RNA was extracted from cells or tissues using the total RNA extraction kit (Life-iLab, AN51L518), and then reverse transcription was performed using oligonucleotide (dT) and reverse transcriptase (Vazyme, R333). Real-time PCR was performed using SYBR qPCR Master Mix (Vazyme, Q711). The relative expression levels of mRNA were calculated using the comparative Ct method (RQ = 2 − ΔΔCt). The PCR primers for murine HSPA8, IL-1β, IL-18, IL-6, TNF-α, GAPDH, and human HSPA8 were synthesized by Sangon (Shanghai, China), and the primer sequences are shown in additional file 1.

### Immunohistochemical staining

After euthanizing the mice, the spinal cords were harvested following cardiac perfusion and immersed in 4% paraformaldehyde (PFA) for fixation for at least 48 h. Subsequently, the tissue was embedded, sectioned, dewaxed, and hydrated, and then stained using the hematoxylin-eosin (H&E) staining kit (Solarbio, G1120) and the myelin staining kit (Luxol Fast Blue (LFB)-tartrazine method) (Beyotime, C0631S). After staining, the sample was fixed with neutral resin and observed and photographed under an inverted microscope (Eclipse Ti-U, Nikon, Japan). ImageJ software (NIH, Bethesda, MD, USA) was used for image analysis.

### Immunofluorescence and co-localization analysis

Spinal cord sections (5 µm) were deparaffinized, subjected to antigen retrieval (citrate buffer, pH 6.0), permeabilized with 0.2% Triton X-100, and blocked with 5% BSA. Sections were incubated overnight at 4°C with primary antibodies against MBP, NF, or Iba1, followed by fluorochrome-conjugated secondary antibodies (FITC/Cy3) and DAPI nuclear staining.

For co-localization, sections were double-stained with rabbit anti-HSPA8 and mouse anti-Iba1, anti-GFAP, or anti-NeuN, then with Cy3 anti-rabbit and FITC anti-mouse secondary antibodies. Images were acquired by confocal microscopy and quantified using ImageJ.

### ASC speck detection

Cells on coverslips were PMA-differentiated, transfected, and activated. After fixation, permeabilization, and blocking, cells were incubated with anti-ASC antibody overnight at 4 °C, then fluorescent secondary antibody, and DAPI. ASC specks were observed under fluorescence microscope and quantified using ImageJ.

### Molecular simulation and docking

Download the HSPA8 protein (source: human PDB ID 3LDQ) as the receptor and the ASC protein (source: human, PDB ID 2KN6) as the ligand from the Uniprot database. Use ZDOCK (website: ZDOCK Server: An automatic protein docking server (wenglab.org)) for global docking, score based on the scoring function, output the top 10 binding conformations, and optimize them.

### Statistical analysis

Data are presented as mean ± SD. All experiments were repeated at least three times. Sample size (*n*) refers to biological replicates. Two-group comparisons were analyzed by unpaired two-sided Student’s *t*-test; multiple groups by one-way ANOVA with Tukey’s post hoc test; EAE clinical scores by two-way repeated-measures ANOVA with Bonferroni post hoc test. *P* < 0.05 was considered significant. SPSS 24 and GraphPad Prism 8 were used.

## Supplementary information

Supplementary Figure legends

Supplementary Figure 1

Supplementary Figure 2

Supplementary Figure 3

additional file 1

Orginal Western Blot Figures

## Data Availability

The data sets used and/or analyzed during the current study are available from the corresponding author on reasonable request.
