# Supplementary material for: Inhibition of HSPA8 alleviates experimental autoimmune encephalomyelitis via dual modulation of NLRP3 inflammasome activation: suppressing both NF-κB–mediated priming and ASC-dependent assembly
Source: Cell Death Discov. 2026 Jun 24;12:358. doi: 10.1038/s41420-026-03215-7 (PMC13547273; doi:10.1038/s41420-026-03215-7)
Supplement: Supplementary file 1 [file 41420_2026_3215_MOESM1_ESM.docx]

**Supplementary Fig. 1 Immunofluorescence co-staining of HSPA8 with CNS cell markers in spinal cord sections from control and EAE mice.** Representative immunofluorescence images showing HSPA8 co-staining with (A) Iba1, (B) GFAP, or (C) NeuN in spinal cord sections from control and EAE mice (n = 6). One‑way ANOVA with Tukey's post hoc test.

**Supplementary Fig.2 HSPA8 knockdown attenuates pyroptosis in THP‑1 cells and BMDMs.** Cells transfected with siNC or siHSPA8 were primed with LPS and stimulated with nigericin. LDH release into the culture supernatant was measured and expressed as a percentage of total LDH content. (A) THP‑1 cells (n=6). (B) BMDMs (n=6). Data are mean ± SD. *^***^P* < 0.001, vs. CON. *^###^P* < 0.001, vs. LPS+ Nig (siNC). One‑way ANOVA with Tukey's post hoc test.

**Supplementary Fig.3 siRNA‑resistant HSPA8 rescue confirms the target specific effect of HSPA8 knockdown on NLRP3 inflammasome activation.** THP-1 cells were co-transfected with siNC or siHSPA8 and empty vector or siRNA-resistant HSPA8 expression plasmid (HSPA8-Rescue). After LPS pretreatment and nigericin stimulation, the activation of the NLRP3 inflammasome was evaluated. (A) ELISA was used to detect the secretion of IL-1β and IL-18 in the supernatant (n = 6). (B) Western blotting was performed to analyze the expression of cleaved caspase-1 (p20) in the supernatant and pro-IL-1β, NLRP3, and HSPA8 in the cell lysate (n = 6). Data are presented as mean ± SD. *^**^P* < 0.01, *^***^P* < 0.001, vs. siNC+ Vector. *^#^P* < 0.05, *^##^P* < 0.01, *^###^P* < 0.001, vs. siHSPA8+Rescue. One-way ANOVA with Tukey's post hoc test was used.
