# Supplementary material for: Inhibition of HSPA8 alleviates experimental autoimmune encephalomyelitis via dual modulation of NLRP3 inflammasome activation: suppressing both NF-κB–mediated priming and ASC-dependent assembly
Source: Cell Death Discov. 2026 Jun 24;12:358. doi: 10.1038/s41420-026-03215-7 (PMC13547273; doi:10.1038/s41420-026-03215-7)
Supplement: Supplementary file 2 [file 41420_2026_3215_MOESM6_ESM.docx]

**Western blots images of Figure**

Western blots images of Figure 1B

Western blots images of Figure 1D

Western blots images of Figure 3D

Western blots images of Figure 4B

Western blots images of Figure 4C

Western blots images of Figure 4E-THP-1

Western blots images of Figure 4E-BMDM

Western blots images of Figure 4E-BV-2

Western blots images of Figure 5C

Western blots images of Figure 5D

Western blots images of Figure 6C

Western blots images of Figure 6F

Western blots images of Figure 7A

Western blots images of Figure 7B

Western blots images of Figure 7D

Western blots images of Figure 7E

Western blots images of Supplementary Fig.3B
